# Chemoprevention with the metabolism modifying drugs dichloroacetate and metformin in the Li-Fraumeni Syndrome model, *Trp53^+/-^* mice

**DOI:** 10.1186/2049-3002-2-S1-P10

**Published:** 2014-05-28

**Authors:** Anneke C Blackburn, Melissa Rooke, Yiming Li, Jane E Dahlstrom, Philip G Board

**Affiliations:** 1 John Curtin School of Medical Research, Australian National University, Canberra, ACT, Australia; 2 Department of Anatomical Pathology, The Canberra Hospital, Woden, ACT, Australia; 3 ANU Medical School, Australian National University, Canberra, ACT, Australia

## Background

While genetic testing for familial cancer has excelled, the prevention options for those carrying high risk alleles have not. Altered bioenergetics is now acknowledged as a hallmark of cancer, and several very safe drugs are available that can target this phentoype. Dichloroacetate (DCA) inactivates pyruvate dehydrogenase kinase, resulting in activation of pyruvate dehydrogenase, reduced lactic acid production and increased mitochondrial activity. Metformin, a type 2 diabetes treatment which activates AMPK, thereby inhibiting mTOR, has unambiguously been demonstrated to reduce the risk of many cancer types in diabetics. We have tested these drugs as chemopreventive agents against the mammary tumours that occur in the BALB/c-*Trp53^+/-^* mouse spontaneous tumour model.

## Materials and methods

Breast cancer cell lines were examined for cell viability after DCA and/or metformin treatment *in vitro* (neutral red uptake assay). Four groups of female BALB/c-*Trp53^+/-^* mice were given distilled water (n=75), DCA (1.5 g/L in drinking water, ~180 mg/ kg/day, n=53), metformin (0.25 g/L in drinking water, ~30 mg/kg/day, n=61) or DCA+metformin (n=51) from 8 weeks of age, and monitored for tumour development over 78 weeks, and Kaplan-Meier survival analysis was performed.

## Results

*In vitro*, DCA (1-5 mM) and metformin (30-300 uM), alone or combined, significantly inhibited breast cancer cell growth. *In vivo*, the overall tumour-free survival curves for BALB/c-*Trp53^+/-^* mice were not significantly different between treatment groups, suggesting that metformin does not reduce cancer risk in non-diabetics. However, analysis of mammary tumours alone found that DCA reduced the number and increased their latency (28.0% vs 20.8% of mice with mean latency of 55.0 vs 63.8 weeks, untreated vs DCA respectively), whereas metformin had no effect (26.2% of mice, mean latency 54.7 weeks). DCA appeared to eliminate the early onset mammary tumours (latency <52 weeks, p=0.02), while not affecting the occurrence of longer latency tumours. In contrast, the two drug combination had worse outcomes for tumour development, (35.3% of mice, latency 48.8 weeks, p<0.02 compared to DCA alone). Preliminary western blotting results in MDA-MB-468 breast cancer cells found that DCA could block the activation of AMPK by metformin, indicating the potential for drug interactions.

## Conclusions

DCA and metformin act differently in the fully transformed cancer cell (growth inhibition) compared to the preneoplastic cell (survival/growth advantage). Examination of the mammary tumour that were“missing” to explain their sensitivity to DCA prevention is underway.

